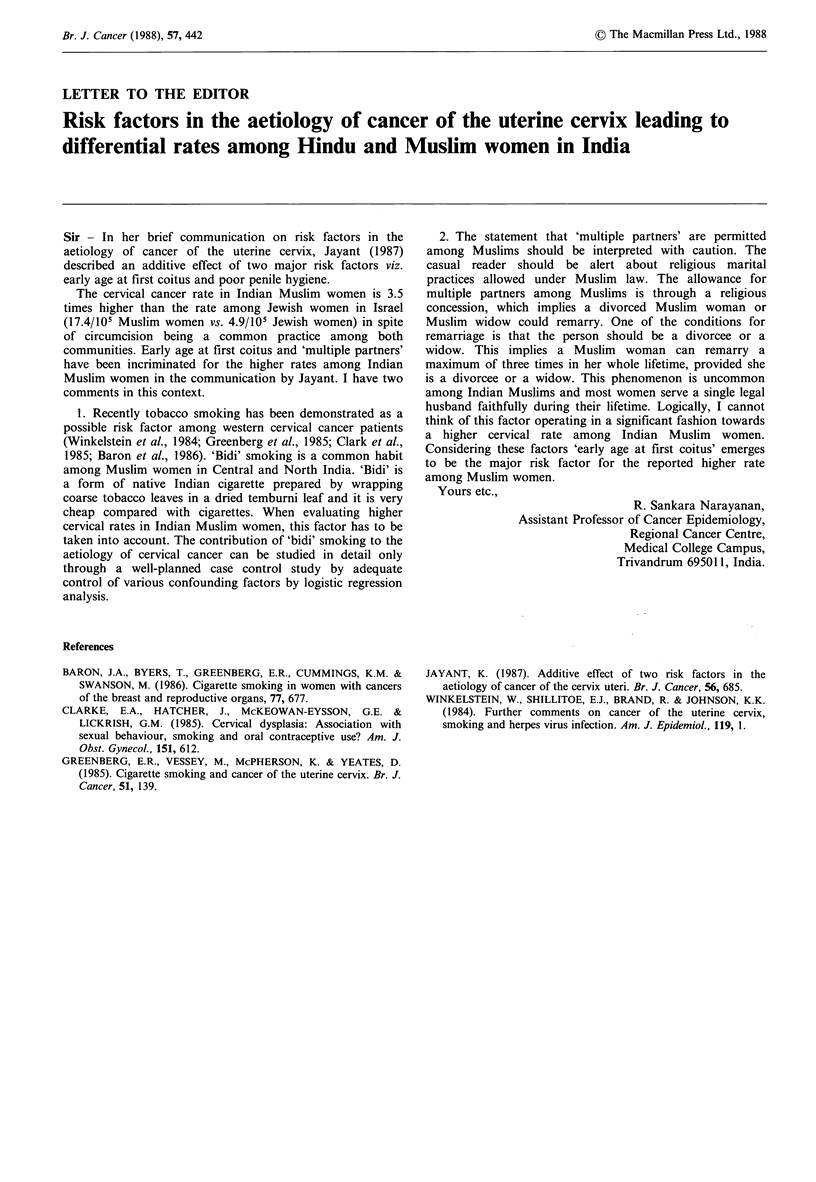# Risk factors in the aetiology of cancer of the uterine cervix leading to differential rates among Hindu and Muslim women in India.

**DOI:** 10.1038/bjc.1988.101

**Published:** 1988-04

**Authors:** R. S. Narayanan


 © The Macmillan Press Ltd., 1988

LETTER TO THE EDITOR

### Risk factors in the aetiology of cancer of the uterine cervix leading to differential rates among Hindu and Muslim women in India

**Sir** – In her brief communication on risk factors in the aetiology of cancer of the uterine cervix, Jayant (1987) described an additive effect of two major risk factors *viz.* early age at first coitus and poor penile hygiene.

The cervical cancer rate in Indian Muslim women is 3.5 times higher than the rate among Jewish women in Israel ($17.4/10^5$ Muslim women *vs.* $4.9/10^5$ Jewish women) in spite of circumcision being a common practice among both communities. Early age at first coitus and 'multiple partners' have been incriminated for the higher rates among Indian Muslim women in the communication by Jayant. I have two comments in this context.

1. Recently tobacco smoking has been demonstrated as a possible risk factor among western cervical cancer patients (Winkelstein *et al.*, 1984; Greenberg *et al.*, 1985; Clark *et al.*, 1985; Baron *et al.*, 1986). 'Bidi' smoking is a common habit among Muslim women in Central and North India. 'Bidi' is a form of native Indian cigarette prepared by wrapping coarse tobacco leaves in a dried temburni leaf and it is very cheap compared with cigarettes. When evaluating higher cervical rates in Indian Muslim women, this factor has to be taken into account. The contribution of 'bidi' smoking to the aetiology of cervical cancer can be studied in detail only through a well-planned case control study by adequate control of various confounding factors by logistic regression analysis.

2. The statement that 'multiple partners' are permitted among Muslims should be interpreted with caution. The casual reader should be alert about religious marital practices allowed under Muslim law. The allowance for multiple partners among Muslims is through a religious concession, which implies a divorced Muslim woman or Muslim widow could remarry. One of the conditions for remarriage is that the person should be a divorcee or a widow. This implies a Muslim woman can remarry a maximum of three times in her whole lifetime, provided she is a divorcee or a widow. This phenomenon is uncommon among Indian Muslims and most women serve a single legal husband faithfully during their lifetime. Logically, I cannot think of this factor operating in a significant fashion towards a higher cervical rate among Indian Muslim women. Considering these factors 'early age at first coitus' emerges to be the major risk factor for the reported higher rate among Muslim women.

Yours etc.,

R. Sankara Narayanan,
Assistant Professor of Cancer Epidemiology,
Regional Cancer Centre,
Medical College Campus,
Trivandrum 695011, India.

**References**

BARON, J.A., BYERS, T., GREENBERG, E.R., CUMMINGS, K.M. & SWANSON, M. (1986). Cigarette smoking in women with cancers of the breast and reproductive organs, **77**, 677.

CLARKE, E.A., HATCHER, J., McKEOWAN-EYSSON, G.E. & LICKRISH, G.M. (1985). Cervical dysplasia: Association with sexual behaviour, smoking and oral contraceptive use? *Am. J. Obst. Gynecol.*, **151**, 612.

GREENBERG, E.R., VESSEY, M., McPHERSON, K. & YEATES, D. (1985). Cigarette smoking and cancer of the uterine cervix. *Br. J. Cancer*, **51**, 139.

JAYANT, K. (1987). Additive effect of two risk factors in the aetiology of cancer of the cervix uteri. *Br. J. Cancer*, **56**, 685.

WINKELSTEIN, W., SHILLITOE, E.J., BRAND, R. & JOHNSON, K.K. (1984). Further comments on cancer of the uterine cervix, smoking and herpes virus infection. *Am. J. Epidemiol.*, **119**, 1.